# Data of range of thoracic kyphosis and lumbar lordosis between line and staff personnel of armed forces in Kermanshah, Iran

**DOI:** 10.1016/j.dib.2022.108194

**Published:** 2022-04-20

**Authors:** Mohammad Bagher Shamsi, Korosh Veisi, Luckman Karimi, Ameneh Safari

**Affiliations:** a School of Rehabilitation Sciences, Kermanshah University of Medical Sciences, Kermanshah, Iran; b Asiastant Professor, Department of Physical Education and Sport Sciences, Sanandaj Branch, Islamic Azad University, Sanandaj, Iran.; c Physical education teacher of Saqqez education office, Ministry of education, Kurdistan province, Iran.; d Research Management Office, School of Allied Medical Sciences, Kermanshah University of Medical Sciences, Kermanshah, Iran

**Keywords:** Thoracic kyphosis, Lumbar lordosis, Line and staff personnel, Armed forces

## Abstract

Two hundred people of the line (new and experienced) and staff (new and experienced) armed forces consisted of the samples. Using a flexible ruler, spinal curves (thoracic kyphosis and lumbar lordosis) were measured. The biggest kyphosis and lordosis values were seen in experienced staff and experienced line groups and the smallest kyphosis and lordosis values were seen in new staff and experienced staff groups, respectively. One-way repeated-measures analysis of variance (ANOVA) was carried out on the thoracic kyphosis and lumbar lordosis measures of the line and the staff groups to assess differences between groups and the post hoc test was carried out to define the group that was significantly distinct from the others.

## Specifications Table

**Table:** 

Subject	Health and medical sciences
Specific subject area	Orthopaedics, Sports Medicine and Rehabilitation
Type of data	Text, Tables, Excel workbook
How data were acquired	The thoracic kyphosis and lumbar lordosis were measured using a flexible ruler.
Data format	Raw and Analyzed
Description of data collection	For measuring the kyphosis, participants were asked to stand comfortablly. Spinous processes of the 2nd and 12th thoracic vertebrae (T2 and T12) were determined. A flexible ruler was placed on the thoracic curve covering the two marked points. Keeping this position, a second coworker marked the adjacent points to T2 and T12 spinous processes on the ruler. The curve was drawn and those two points were reflected on the paper. A straight line was drawn connecting T2 and T12 points. The length of this line and the distance between the deepest point of the curve and the line were measured. Using them and the equation below, the magnitude of the angle of the thoracic curve was measured. *θ =*4 Arctang (2*h*/*l*) The same process was done for the Lordosis measurement unless the spinous processes of L1 and L5 were marked and measured instead.
Data source location	Institution: Kermanshah University of Medical Sciences City: Kermanshah Country: Iran
Data accessibility	‘With the article’

## Value of the Data

• The data provide insights into the prevalence of kyphosis and lordosis in staff personnel of the armed forces.
• Researchers and health policymakers and military commanders can benefit from these data to propose new physical behaviors for staff personnel health.
• This study can be one of the first steps in understanding the status of spinal curves in personnel of the armed forces. Next studies from other parts of the world help to have a better insight into a global status.
• This data can be linked with other available datasets, to provide insight into what factors influence the prevalence of kyphosis and lordosis in staff.

## 1 Data Description

The data presented in this paper were tables and datasets. The military and security forces of nations depend partly on army personnel's physical performance and health. Many factors can affect their physical health and especially their postural alignment such as work conditions, the style of doing physical works, bad and sustained postures, wrong movement patterns, doing military operations, parades, overweight, and so on [1,2]. So it is necessary to assess these personnels regularly about their health including physical health and posture [2]. The data obtained are presented in an excel workbook. The main characteristics of the subjects was presented in Table 1. In addition, the difference in kyphosis and lordosis between the two groups and the normal range of thoracic kyphosis and lumbar lordosis in staff and line personnel were presented in Tables 2 and 3, respectively.

**Table 1:** Participants characteristics.

	Staff groups		Line groups	
	New staff group(*n* = 50)	Staff group with more than 5 year of experience (*n* = 50)	New line group(*n* = 50)	Line group with more than 5 year of experience (*n* = 50)
Age(year)	24.10 ± 1.95	28.52 ± 1.31	22.18 ± 2.13	28.60 ± 2.11
Length (cm)	177.94 ± 5.79	177.60 ± 6.09	176.86 ± 5.07	177.92 ± 4.58
Weight (kg)	75.82 ± 11.17	81.56 ± 10.23	75.46 ± 10.37	84.6 ± 9.64
Thoracic kyphosis	38.73 ± 7.93	43.50 ± 7.98	38.87 ± 7.98	39.53 ± 7.18
Lumbar lordosis	39.94 ± 10.49	36.48 ± 8.16	39.88 ± 8.06	40.08 ± 10.48

Unite of lordosis and kyphosis = Degree.

**Table 2:** *P*-value for difference in kyphosis and lordosis between the two groups.

Groups	New staff group	Staff group with more than 5 year of experience	New line group	Line group with more than 5 year of experience
New staff group	Kyphosis P = 1 Lordosis P = 1	Kyphosis P = 0.003* Lordosis P = 0.066	Kyphosis P = .0932 Lordosis P = 0.975	Kyphosis P = 0.608 Lordosis p = 0.938
Staff group with more than 5 year of experience	Kyphosis P = 0.003* Lordosis P = 0.066	Kyphosis P = 1 Lordosis P = 1	Kyphosis P = 0.003* Lordosis P = 0.071	Kyphosis P = 0.012* Lordosis P = 0.056
New line group	Kyphosis P = 0.932 Lordosis P = 0.975	Kyphosis P = P = 0.003* Lordosis P = 0.071	Kyphosis P = 1 Lordosis P = 1	Kyphosis P = 0.669 Lordosis P = 0.913
Line group with more than 5 year of experience	Kyphosis P = 0.608 Lordosis P = 0.938	Kyphosis P = 0.003* Lordosis P = 0.056	Kyphosis P = 0.669 Lordosis P = 0.913	Kyphosis P = 1 Lordosis P = 1

Unite of lordosis and kyphosis = Degree; Unite of length of curve = Centimeter.

**Table 3:** Normal range of thoracic kyphosis and lumbar lordosis in staff and line personnel.

Groups	Mean ± SD	Range
Thoracic kyphosis curve Staff groups Lumbar lordosis curve	42.54 ± 8.36	25.83–59.25
	38.21 ± 9.51	19.18–57.23

Thoracic kyphosis curve Line groups Lumbar lordosis curve	40.28 ± 7.47	25.34–55.21
	39.98 ± 9.30	21.38–58.58

Unite of lordosis = Degree; Unite of length of curve = Centimeter.

Demographic characteristics and the amount of spinal curves for 200 subjects (50 in each group) are presented in Table 1.

## 2 Experimental Design, Materials and Methods

### 2.1 Instrument of data collection

The study design was descriptive and cross-sectional. Army staff working in Kermanshah, a city in the west of Iran was the population of the study. Four groups were defined: 1- Experienced line worker group with more than 5 years period of working (EL), 2- New line worker group with less than one year period of working (NL), 3- Experienced staff worker group with more than 5 years period of working (ES), 4-. New staff worker group with less than one year period of working (NS). As no related study on army staff was found, the sample size was defined as 10% of the total 2000 population (200 people). This number included 50% for the line and 50% for the staff group. Using the available sampling method, from all 4 garrisons located in Kermanshah, subjects were chosen for all groups. The sampling rate for each garrison was in proportion to its population. Using a flexible ruler, thoracic kyphosis and lumbar lordosis for all subjects were measured as it is explained in our previous paper [3].

One-way analysis of variance (ANOVA) was carried out on the thoracic kyphosis and lumbar lordosis measures of line and staff groups to assess differences between groups and a post hoc test was carried out to define the group that was significantly distinct from the others.

### 2.2 Data presentation

The biggest kyphosis and lordosis values (in degree) were seen in the ES (43.50) and the EL (40.08) and the smallest kyphosis and lordosis values were seen in the NS (38.73) and the ES (36.48) groups, respectively. With the one-way repeated-measures ANOVA, we found a difference between thoracic kyphosis in the experienced staff workers group and the other three groups (*p* < 0.05). There was no difference in lumbar lordosis between groups (*p* < 0.05) (Table 2). As ± two standard deviations from the mean were defined as “normal,” [4] (which includes 95% of the population), normal ranges of thoracic kyphosis and lumbar lordosis in the subjects in the four garrisons were calculated and are presented in Table 3.

## Ethics Considerations

Ethical approval was obtained from Baghyatollah Medical Sciences University. Written informed consent was obtained from study participants and their identity was kept confidential.

## CRediT authorship contribution statement

**Mohammad Bagher Shamsi:** Conceptualization, Methodology, Formal analysis, Writing – original draft, Writing – review & editing. **Korosh Veisi:** Conceptualization, Methodology, Writing – review & editing, Methodology, Formal analysis, Supervision, Funding acquisition. **Luckman Karimi:** Methodology, Formal analysis, Writing – original draft, Writing – review & editing. **Ameneh Safari:** Writing – review & editing, Data curation, Formal analysis.

## Declaration of Competing Interest

The authors declare that they have no known competing financial interests or personal relationships which have or could be perceived to have influenced the work reported in this article.

## Data Availability

supplementary file (Original data) (www.targetsite.edu/datasets/dataset1).

## Appendix Supplementary Materials

Image, application 1
